# A comparison of breast cancer survival across different age groups: a multicentric database study in Penang, Malaysia

**DOI:** 10.4178/epih.e2021038

**Published:** 2021-05-25

**Authors:** King Fang Tan, Farzaana Adam, Hasmah Hussin, Noor Mastura Mohd Mujar

**Affiliations:** 1 Institute Latihan Kementerian Kesihatan Malaysia Sultan Azlan Shah, Ministry of Health Malaysia, Perak, Malaysia; 2 Lifestyle Science Cluster, Advanced Medical and Dental Institute, Universiti Sains Malaysia, Penang, Malaysia; 3 Penang State Health Department, Ministry of Health Malaysia, Penang, Malaysia; 4 Women Cancer Unit (UNITA), Clinical Section of Advanced Medical and Dental Institute, Universiti Sains Malaysia, Penang, Malaysia; 5 Regenerative Medicine Cluster, Advanced Medical and Dental Institute, Universiti Sains Malaysia, Penang, Malaysia

**Keywords:** Breast neoplasms, Survival, Age groups, Prognosis, Asian continental ancestry group

## Abstract

This study compared breast cancer survival and the prognostic factors across different age groups of women in Penang, Malaysia. Data on 2,166 women with breast cancer who had been diagnosed between 2010 and 2014 were extracted from the Penang Breast Cancer Registry and stratified into 3 age groups: young (< 40 years old), middle-aged (40-59 years old), and elderly (≥ 60 years). The overall and relative survival rates were calculated using the life table method, median survival time was calculated using the Kaplan-Meier method, and comparisons between groups were conducted using the log-rank test. Prognostic factors were analyzed using a Cox proportional hazards model. The 5-year overall and breast cancer-specific survival rates for women with breast cancer in Penang were 72.9% and 75.2%, with a mean survival time of 92.5 months and 95.1 months, respectively. The 5-year breast cancer-specific survival rates for young, middle-aged, and elderly women were 74.9%, 77.8%, and 71.4%, respectively, with a mean survival time of 95.7 months, 97.5 months, and 91.2 months. There was a significant difference in breast cancer survival between age groups, with elderly women showing the lowest survival rate, followed by young and middle-aged women. Disease stage was the most prominent prognostic factor for all age groups. Survival rates and prognostic factors differed according to age group. Treatment planning for breast cancer patients should be age-specific to promote better cancer care and survival.

## INTRODUCTION

Breast cancer is the most commonly occurring cancer among women, and is responsible for a considerable number of cancer-related deaths globally [1]. It is also the most common cancer in Malaysia, accounting for 34.1% of all cancers nationwide [2]. In 2018, at least 7,593 women in Malaysia had breast cancer, and as many as 2,894 of them died from breast cancer that year [3]. In the latest report by the Malaysian National Cancer Registry, there was an increase in breast cancer incidence from 18,206 between 2007 and 2011 to 21,634 between 2012 and 2016. In addition, the report showed an increasing number of women being diagnosed with breast cancer at younger ages and at more advanced cancer stages compared to the previous report [2,4]. Moreover, the Malaysian Study on Cancer Survival reported a dismal 66.8% breast cancer survival rate [5], which is much lower than that reported for other Asian countries such as Japan (96.2%) [6], Korea (92.6%) [7], and Singapore (79.0%) [8].

To date, various studies have been conducted on breast cancer survival and prognostic factors. Significant prognostic factors include age, ethnicity, tumor type, tumor size, number of lymph nodes affected, disease stage, hormone receptor status, and human epidermal growth factor receptor-2 status. In a recent Malaysian study, the disease stage and ethnicity were independent prognostic factors affecting breast cancer survival, with advanced cancer stages and being of the Malay ethnicity corresponding to a significantly lower survival rate [5]. Even though the relationship between breast cancer survival and age at diagnosis has been explored in many studies, information on survival rates across different age groups remains unclear, especially in Asian contexts. Furthermore, there have been conflicting results between published studies, with some studies reporting that young women diagnosed with breast cancer had the lowest survival rate [9] and others claiming that the survival rate was lowest for elderly patients [10]. Other studies have reported that one’s age at diagnosis had no significant effect on breast cancer survival [11]. In addition, most of the studies were conducted in single-centre settings, with most of the institutions under study being concentrated in the capital city of Kuala Lumpur. Thus, past study findings may not be generalisable to the Malaysian population. Therefore, this multicentre study was conducted to compare breast cancer survival rates between women in different age groups in Penang, Malaysia.

## MATERIALS AND METHODS

### Location, population, and study design

**Figure 1.**
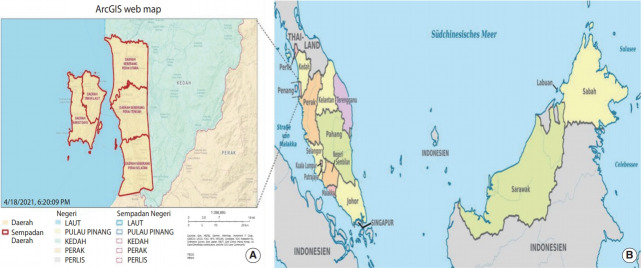
(A) Geographical location of the Penang Cancer Registry’s population [12], (B) geographical location of Penang in Malaysia [13].

**Figure 2.**
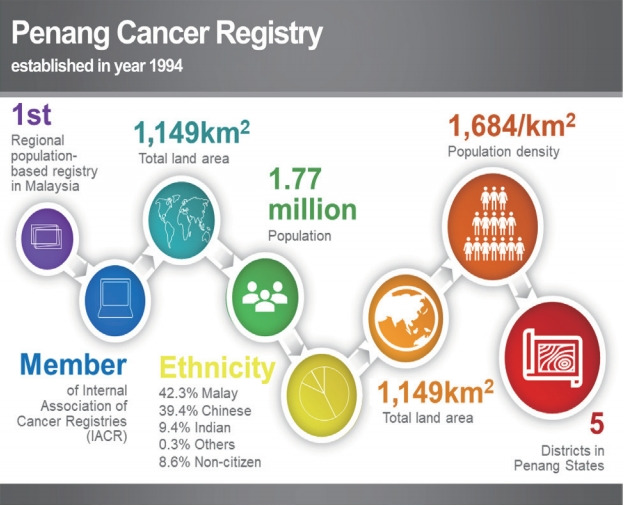
Composition of the Penang Cancer Registry’s population demographics [14].

This study was conducted in Penang State, Malaysia. Penang is a state located in the northern part of the Malaysian peninsula that consists of an island and a strip of mainland, with a total land area of 1,149 km^2^ (Figure 1) [12,13]. The state has a population of 1.77 million people with a population density of approximately 1,684 people per square kilometre as of 2018. The population distribution is 42.3% Malay, 39.4% Chinese, 9.4% Indian, and 0.3% other ethnicities, with 8.6% of the population being non-Malaysian (Figure 2) [14].

Secondary data obtained from the Penang Cancer Registry under the Penang State Health Department, Malaysia, were used in this study. All 2,166 women Malaysian citizens who had a primary breast cancer diagnosis between January 1, 2010, and December 31, 2014, were universally sampled. All subjects were then stratified into 3 groups by age: young women (< 40 years), middle-aged women (40-59 years), and elderly women (≥ 60 years). Survival time (in months) was calculated from the time of breast cancer histopathological diagnosis to the time of the event (death of any cause) or last follow-up date, up to December 31, 2019 (censored). Subjects’ identification card numbers were used to cross-check survival status with the National Registration Department.

### The Penang Cancer Registry database

The Penang Cancer Registry is a regional population-based registry managed by the Penang State Health Department, Malaysia, that was established in 1994. The Penang Cancer Registry is also listed as a member of the International Association of Cancer Registries. The cancer registry receives notifications from all data sources used in government and private health facilities using a standardised notification form. The registry records socioepidemiological details and important histological and clinical data for all cancer cases registered in Penang regarding both diagnosis and treatment.

### Data quality

Cancer registration data were obtained from a combination of sources using passive and active case detection, including from notifications by medical personnel, pathology records, hospital discharges records, mortality data from the National Registration Department of Malaysia, and hospital death notifications. In this study, all deaths for which notifications were received were verified for authenticity with the National Registration Department. All breast cancer cases were verified for authenticity by registry staff before they were incorporated into the database. All histological types were confirmed by a pathologist for either histological or cytological/haematological confirmation. Patients’ age was reported specifically for this study.

### Instruments

A predesigned form was used to guide data extraction in this study. The data extracted included socio-demographic characteristics such as age at diagnosis, ethnicity, and clinical information such as disease stage, histological type, date of diagnosis, date of death, vital status (deceased or living), and treatment type (surgery, chemotherapy, radiotherapy, and hormonal therapy).

### Statistical analysis

IBM SPSS version 24 (IBM Corp., Armonk, NY, USA) was used for data analysis. For descriptive statistics, the categorical variables were presented as frequency and percentage (%). Normally distributed variables were presented as mean and standard deviation, whereas abnormally distributed variables were presented as the median and interquartile range. In addition, the Pearson chi-square test was performed to compare patients’ characteristics by age group. The 1-year, 3-year, and 5-year overall survival rates and cause-specific survival rates of the 3 age groups of young, middle-aged, and elderly women with breast cancer were calculated with the actuarial life-table method. The Kaplan-Meier method was used to calculate the median survival time, while differences in survival rates were determined using the log-rank test. Lastly, the prognostic factors for survival time were analysed using a Cox proportional hazards model. Due to possible competing risks, survival analysis is presented in terms of both all-cause death and breast-cancer-specific death in this study. The final model was tested for the proportional-hazards assumption, which was found to be met. All possible 2-way interactions between variables were checked and interactions were found between age group and ethnicity, disease stage, and histological type. Therefore, age group was identified as a confounding factor in this study, and separate models were constructed for each age group. A p-value below 0.05 was considered to indicate statistical significance.

### Ethics statement

Ethical approval was obtained from the Human Research Ethics Committee, Universiti Sains Malaysia (USM/JEPeM/18090444) and Medical Research and Ethics Committee, Ministry of Health Malaysia (NMRR-18-2461-42933 [IIR]).

## RESULTS

Out of a total of 2,222 breast cancer cases, 36 non-Malaysian citizens and 20 man patients were excluded. Thus, 2,166 cases were included in the final analysis. The socio-demographic and clinical characteristics of women with breast cancer were summarized based on the three age groups (Table 1). More than half (54.4%) of the subjects were middle-aged women, and the majority were Chinese (62.9%), followed by Malay (27.0%), Indian (9.0%), and others (1.1%). The median age at diagnosis for all women was 55 years old, with a range of 19 years to 93 years. In addition, the individual median ages for the young, middle-aged, and elderly groups were 36, 50, and 67, respectively.

The disease stage was only reported for 57.1% of the women, most of whom had early-stage disease (36.1% of the total number of women). Infiltrating ductal carcinoma (IDC) was the most common histological type. Approximately 3 in 4 women had IDC, and the trend was similar across all three age groups. The 4 other common histological types included ductal carcinoma in situ (6.8%), lobular carcinoma (3.3%), mucinous carcinoma (1.9%), and medullary carcinoma (2.3%). More than half of the women received treatment that involved surgery (61.0%), with 36.1% receiving surgery only and 25.0% receiving surgery with oncologic therapy. Only a small percentage of women (13.5%) received only oncologic therapy, meaning a combination of one or more therapies consisting of chemotherapy, radiotherapy, and hormonal therapy. The characteristics of subjects according to age group are listed in Table 1.

### Survival analysis

**Figure 3.**
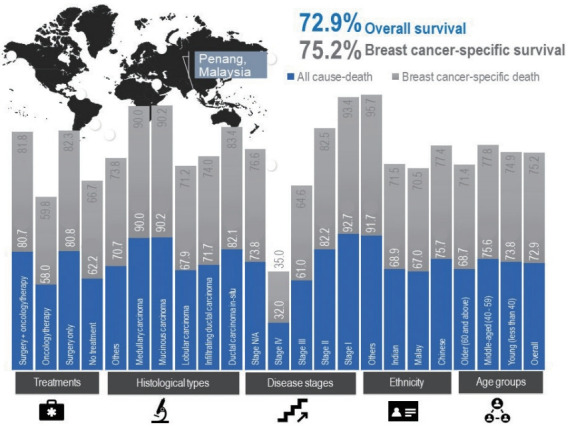
The 5-year survival rate (%) by prognostic factors. N/A, not available.

**Figure 4.**
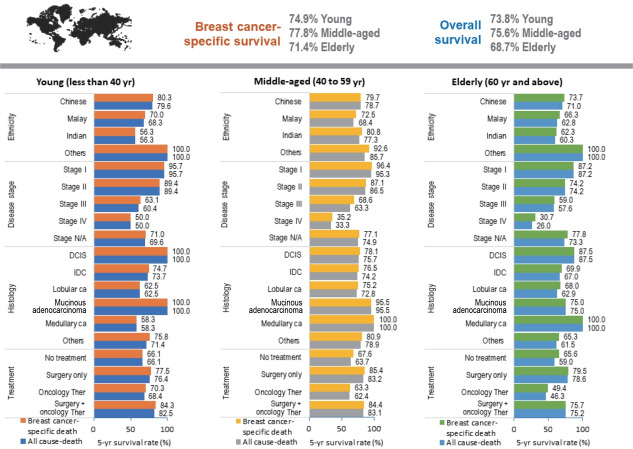
The 5-year survival rate (%) by age groups. N/A, not available; DCIS, ductal carcinoma in situ; IDC, infiltrating ductal carcinoma; ca, carcinoma; Ther, therapy.

At the end of the follow-up period for this study, 1,580 (72.9%) women had survived breast cancer, of whom 161 were young (7.4%), 891 were middle-aged (41.1%), and 528 were elderly (24.4%). A total of 586 (27.1%) deaths were recorded, of which 525 (24.2%) were due to breast cancer. The 5-year overall and breast cancer-specific survival rates for women with breast cancer in this study were 72.9% and 75.2%, with mean survival times of 92.5 months and 95.1 months, respectively. Between the age groups, the 5-year breast cancer-specific survival rates for young, middle-aged, and elderly women were 74.9%, 77.8%, and 71.4%, with mean survival times of 95.7 months, 97.5 months, and 91.2 months, respectively. In this study, only women with stage IV breast cancer had a lower than 50% survival rate 5 years after receiving a diagnosis. Thus, the estimated median survival time for stage IV patients was 32 months for all age groups, 57 months for young women, 33 months for middle-aged women, and 23 months for elderly women. The survival rates of women with breast cancer according to prognostic factors are shown in Figures 3 and 4, respectively.

**Figure 5.**
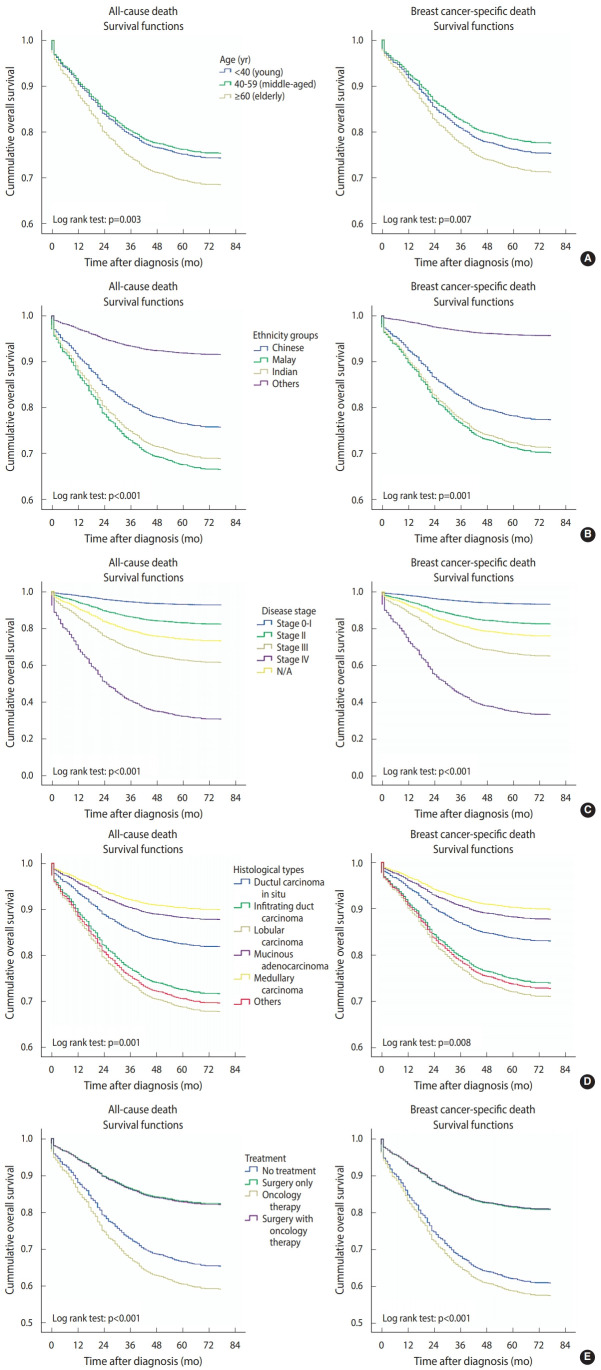
Kaplan-Meier plot according to all-cause death and breast cancer-specific death for (A) age groups, (B) ethnicity groups, (C) disease stage. N/A, not available. (D) histological types, (E) treatment types.

Age at diagnosis was a significant prognostic factor for breast cancer. Survival rates were different for each age group. Of the age groups, elderly women had the lowest survival time and survival rate. Although the survival rate of elderly women was not significantly different from that of young women, it was significantly lower than that of middle-aged women (Figure 5).

Univariable and multivariable Cox regression analysis for all-cause death and breast cancer-specific death is shown in Table 2. Both analyses showed that elderly women, women of Malay ethnicity, women with stage II, III, and IV breast cancer, women with histological findings of ductal carcinoma or lobular carcinoma, and women who did not receive surgical treatment were more vulnerable to breast cancer-specific death. Disease stage was a prominent prognostic factor, with advanced stages corresponding to the lowest survival rates, while surgery was a protective factor for breast cancer survival.

Univariable and multivariable Cox regression analyses comparing all-cause death and breast cancer-specific death by age are presented in Tables 3 and 4. The results were very similar for both analyses. However, the prognostic factors differed by age. Indian ethnicity and stage IV breast cancer were prognostic factors for young women, while advanced-stage cancer and surgical treatment were prognostic factors for middle-aged women. Meanwhile, advanced-stage cancer, histological findings of ductal carcinoma and lobular carcinoma, and surgical treatment were prognostic factors for the elderly.

## DISCUSSION

### Age differences and survival

In this study, half of the subjects were diagnosed with breast cancer during middle age, and the median age was 55 years old. This contrasts with other Malaysian studies in which the majority of subjects were elderly, with an average age range between 60 years to 64 years old [2,15,16]. In addition, only 10% of women with breast cancer in this study were in the young age group—a lower proportion compared to the 13-15% rate of younger women [15,17] reported in other Malaysian studies. Furthermore, the 5-year survival rate in this study (75.2%) was higher than the rate reported by a national cancer study (66.8%) [5], thus possibly indicating a better survival rate among middle-aged women with breast cancer in the Northern region of Malaysia than among the national population.

Additionally, age at diagnosis was found to be a significant predictor of breast cancer survival, echoing another populationbased study undertaken in Malaysia [9]. However, most studies did not identify the most affected age groups. In contrast, we found that elderly women with breast cancer had the shortest median survival time and the lowest survival rate compared to their younger counterparts. Thus, this shows that elderly women were the most affected by a breast cancer diagnosis and had the lowest survival rate of all 3 age groups. This finding is similar to those of previous population-based studies by Abdullah et al. [10], which compared breast cancer survival rates between patients whose ages at diagnosis were < 50 years and ≥ 50 years, and Nematolahi & Ayatollahi [18], which compared the breast cancer survival rates of young, middle-aged, and elderly women in Iran. Nevertheless, our finding contrasts with another local study conducted in Kelantan, Malaysia, that showed that younger women had a higher excess hazard and lower likelihood of survival than other age groups following a breast cancer diagnosis [9]. However, in this study, we found that age groups were the confounding factors in this study. The age group-specific models used to analyse survival rates in this study revealed that the prognostic factors for different age groups were not the same. Therefore, we suggest using an age group-specific model to analyze survival rates in future studies.

### Age differences, ethnicity, and survival

Similar to the findings of previous studies [15,16,19], ethnicity was a significant prognostic factor affecting the survival of Malaysian women with breast cancer in this study. Of the ethnicities included in the study, Malay women were more likely to be diagnosed at a young age than Chinese women. However, even though Chinese women were more often diagnosed at an elderly age, they had a significantly better survival rate than other ethnicities. A similar finding was reported in other studies conducted in Malaysia [5,19]. Our findings also revealed an impact of ethnicity on breast cancer survival in Malaysia [15]. Regardless of age, Malay women recorded a higher mortality rate than Chinese and Indian women. The high mortality rate among Malay women could be due to the common use of complementary and alternative medicine in Malay culture, which may subsequently result in delayed presentation [20]. Young and elderly Indian women were found to have had significantly higher mortality rates than Chinese women even after the confounders were controlled via stratification in this study. This finding is similar that of a previous study of the Singapore-Malaysia hospital-based breast cancer registry, which reported a moderately high mortality risk among Indian women when compared to Chinese women [19].

Ethnic differences are often linked to differences in socioeconomic status, which further affect patients’ access to medical treatment and, in turn, survivorship. In our study population, Malay women had the lowest average household income, followed by Indian and Chinese women [14]. However, access to medical services in Malaysia is subsidised for all citizens regardless of ethnicity and socioeconomic status. Therefore, socioeconomic status might not directly affect medical access, but other factors such as health beliefs might be related to ethnic disparities in breast cancer survivorship.

### Age differences, disease stage, and survival

Compared to other studies, the women in this study were more likely to be diagnosed with early-stage breast cancer rather than advanced-stage breast cancer [2]. However, there was a high proportion of missing data for the disease stage variable, with data on disease stage missing for 42.9% of the respondents included in our study compared to only 32.7% in national cancer reports [2]. Therefore, there is a chance that the data were missing for advanced-stage breast cancer in this study. The proportion of missing cancer stage data in this study exceeds the 30% maximum theoretically allowable proportion of missing values for analysis. However, after checking the pattern of missing data, no significant pattern was found with regard to the effect of cancer stage on breast cancer-specific death by age group. Thus, we did not exclude this variable from the analysis.

In this study, women with stage IV breast cancer had a much higher median survival time (32 months) compared to the median survival time found by another recent study (6.9 months) [11]. However, the median survival time for stages 0, I, II, and III in this study could not be obtained since they contributed to fewer than 50% of reported deaths. Furthermore, this study showed that disease stage was an important prognostic factor for breast cancer, echoing the findings of other studies [11,17,21]. Overall, the survival rate of women with breast cancer decreased as the disease stage advanced. It was found that women with stage III and stage IV breast cancer had significantly lower survival rates across all 3 age groups. This indicates that advanced breast cancer stages are associated with significant increases in mortality risk regardless of age. This finding is consistent with previous studies [11,17,21]. Nevertheless, a recent study of a district hospital found that survival was poorer for women whose disease stages were unknown, possibly because they were often diagnosed at a much later stage [22].

### Age differences, histological type, and survival

IDC was the most common type of breast cancer across all age groups in this study. Other subtypes, including lobular, mucinous, and medullary carcinoma, were more common in young women, while ductal carcinoma in situ was more prevalent among elderly women. In this study, significantly higher mortality was observed among women with IDC, lobular carcinoma, and other histological types compared to ductal carcinoma in situ. However, the age group-stratified analysis found that the histological subtype only appeared to significantly affect the survival of elderly women. This finding is similar to the results of other studies that found that histological subtype was a prognostic factor for breast cancer. Several studies pointed out that women with mucinous and medullary carcinoma had significantly higher survival rates and longer survival times than those with IDC [23-26]. Generally, these subtypes had a more favourable outcome than IDC since they tend to be associated with smaller tumors [27], less aggressive local invasion [23], and slower disease progress [28]. Another cohort study in the United States reported that mucinous, tubular, and medullary carcinoma were often associated with better outcomes [26]. However, due to limitations with regard to the use of secondary data in this study, some relevant variables such as tumor size, nodule number, tumor grade, and hormonal receptor status (oestrogen receptor/progesterone receptor/HER2) were unavailable, thus preventing a more comprehensive analysis of the data.

### Age differences in treatment and survival

Surgical treatment was the primary treatment for women with breast cancer across all age groups in this study. It was also a significant prognostic factor associated with breast cancer mortality. This study further reinforces the effects of surgical treatment on survival for women with breast cancer across different age groups. In this study, surgical treatment alone and surgical treatment with oncologic therapy were associated with a significant 50% reduction in mortality among women with breast cancer. After stratifying by age group, middle-aged and older women who received surgical treatment with oncologic therapy also had a significantly lower breast cancer mortality—by 57%—which is consistent with the findings of other studies in Malaysia [9,11]. The findings of this study further reinforce that surgical treatment could improve the chances of survival for women with breast cancer, with the benefits appearing to be even greater for middle-aged and elderly women. However, this relationship must be confirmed with randomized trials since survival rates are not just related to age and surgical treatment, and can also be influenced by other disease characteristics.

In this study, oncologic treatment, which includes some combination of chemotherapy, radiotherapy, and hormonal therapy, did not show a significant association with breast cancer survival except for when chemotherapy was the primary treatment for elderly women, which was associated with a significant increase in mortality. However, this effect was not significant after adjustment for other variables. Nevertheless, only a small number of women across all age groups received oncologic treatment in this study, similar to the 8% to 28% uptake recorded in another study [9]. Although these therapies are commonly practised in the treatment of breast cancer, only a few studies have found significant associations between oncologic therapy and breast cancer survival [29,30]. Individually, hormonal therapy was linked to a substantial reduction in the risk of mortality by 3 times when conducted with menopausal women [29]. However, more studies are needed to discover further evidence and to explore the effectiveness of different adjuvant treatments on the survival of breast cancer patients.

This was a retrospective cohort study that retrieved secondary data from a regional cancer registry database, in which data were reported voluntarily by health facilities. Thus, only information available in this registry was included in the analysis, and the high percentage of missing data may have led to some bias. More importantly, essential data, such as the tumour size and grade, lymph node involvement, and hormonal receptor status (oestrogen receptor/progesterone receptor/HER2), were unavailable.

However, despite the limitations, this study was able to highlight several new prognostic factors specific to certain age groups that are crucial for improving the breast cancer management among women. Furthermore, women with breast cancer in this study were classified into 3 groups—young, middle-aged, and elderly—compared to other studies, which largely classified subjects as either pre-menopausal or post-menopausal [9,10]. Thus, our study has additional relevance since it accurately identified the effect of age on survival prognosis. Nevertheless, studies with a larger sample size are needed in the future to encompass other variables such as family history, histopathology, and psychosocial factors to better explore survival in breast cancer patients of different ages.

## CONCLUSION

The study findings point to significant differences in the survival rates of breast cancer patients by age. Elderly women had significantly poorer survival than middle-aged women. Age at diagnosis, ethnicity, disease stage, histological type, and surgical treatment were the significant predictors of breast cancer survival. Elderly women (≥ 60 years), women who were of the Malay ethnicity, women with stages II, III, and IV cancer at diagnosis, and women who did not undergo surgical treatment had a higher risk of mortality. In summary, survival rates and prognostic factors were different in each age group, and elderly women had the lowest likelihood of survival. Based on these findings, treatment planning for women with breast cancer should be age-specified to promote better cancer care and a higher likelihood of survival.

## Figures and Tables

**Table 1.** Characteristics of women with breast cancer by age

Variables		Young (n=218)	Middle-aged (n=1,179)	Elderly (n=769)	Total (n=2,166)
Age at diagnosis (yr)^1^					
	Mean±SD	34.78±4.06	50.19±5.49	68.21±6.73	55.04±12.24
	Median [Min-Max]	36 [19-39]	50 [40-59]	67 [60-93]	55 [19-93)
Ethnicity					
	Chinese	118 (54.1)	708 (60.1)	537 (69.8)	1,363 (62.9)
	Malay	82 (37.6)	355 (30.1)	148 (19.2)	585 (27.0)
	Indian	16 (7.3)	102 (8.7)	76 (9.9)	194 (9.0)
	Others	2 (0.9)	14 (1.2)	8 (1.0)	24 (1.1)
Stage					
	0-I	23 (10.6)	173 (14.7)	94 (12.2)	290 (13.4)
	II	47 (21.6)	261 (22.1)	184 (23.9)	492 (22.7)
	III	28 (12.8)	142 (12.0)	90 (11.7)	260 (12.0)
	IV	18 (8.3)	99 (8.4)	77 (10.0)	194 (9.0)
	Not available	102 (46.8)	504 (42.7)	324 (42.1)	930 (42.9)
Histology					
	Ductal carcinoma in situ	6 (2.8)	74 (6.3)	67 (8.7)	147 (6.8)
	Infiltrating ductal carcinoma	164 (75.2)	932 (79.1)	575 (74.8)	1,671 (77.1)
	Lobular carcinoma	8 (3.7)	37 (3.1)	27 (3.5)	72 (3.3)
	Mucinous adenocarcinoma	7 (3.2)	22 (1.9)	12 (1.6)	41 (1.9)
	Medullary carcinoma	12 (5.5)	28 (2.4)	10 (1.3)	50 (2.3)
	Others	21 (9.6)	86 (7.3)	78 (10.1)	185 (8.5)
Treatment					
	No treatment	56 (25.7)	290 (24.6)	205 (26.7)	551 (25.4)
	Surgery only	72 (33.0)	402 (34.1)	307 (39.9)	781 (36.1)
	Surgery with oncologic therapy	52 (23.9)	327 (27.7)	162 (21.1)	541 (25.0)
	Oncologic therapy^1^	38 (17.4)	160 (13.6)	95 (12.4)	293 (13.5)

Values are presented as number (%). SD, standard deviation; Min, minimum; Max, maximum.

1 A combination of one or more therapies consisting of chemotherapy, radiotherapy, and hormonal therapy.

**Table 2.** Univariable and multivariable Cox regression analysis for prognostic factors of breast cancer related to all-cause death and breast cancer-specific death^1^

Variables		All-cause death				Breast cancer-specific death			
		Crude HR^2^ (95% CI)	p-value	Adjusted HR^3^ (95% CI)	p-value	Crude HR^2^ (95% CI)	p-value	Adjusted HR^3^ (95% CI)	p-value
Age (yr)			0.003		0.001		0.007		0.005
	<40	1.05 (0.79, 1.40)	0.720	0.93 (0.70, 1.23)	0.606	1.12 (0.84, 1.50)	0.450	0.99 (0.73, 1.32)	0.924
	40-59	1.00 (reference)	-	1.00 (reference)	-	1.00 (reference)	-	1.00 (reference)	-
	≥60	1.34 (1.13, 1.59)	0.001	1.36 (1.14, 1.61)	0.001	1.34 (1.12, 1.60)	0.002	1.34 (1.12, 1.61)	0.002
Ethnicity			<0.001		0.014		0.001		0.047
	Chinese	1.00 (reference)	-	1.00 (reference)	-	1.00 (reference)	-	1.00 (reference)	-
	Malay	1.47 (1.23, 1.75)	<0.001	1.27 (1.06, 1.53)	0.011	1.38 (1.14, 1.67)	0.001	1.21 (1.00, 1.48)	0.056
	Indian	1.35 (1.02, 1.77)	0.034	1.25 (0.94, 1.64)	0.120	1.32 (0.99, 1.77)	0.059	1.23 (0.92, 1.64)	0.167
	Others	0.32 (0.08, 1.27)	0.105	0.31 (0.08, 1.25)	0.099	0.17 (0.02, 1.23)	0.079	0.17 (0.02, 1.22)	0.077
Stage			<0.001		<0.001		<0.001		<0.001
	0-I	1.00 (reference)	-	1.00 (reference)	-	1.00 (reference)	-	1.00 (reference)	-
	II	2.54 (1.58, 4.09)	<0.001	2.42 (1.50, 3.90)	<0.001	2.75 (1.68, 4.53)	<0.001	2.61 (1.59, 4.29)	<0.001
	III	6.37 (3.98, 10.20)	<0.001	6.03 (3.76, 9.66)	<0.001	6.20 (3.77, 10.17)	<0.001	5.88 (3.57, 9.66)	<0.001
	IV	15.56 (9.81, 24.67)	<0.001	11.08 (6.91, 17.75)	<0.001	15.99 (9.85, 25.97)	<0.001	11.41 (6.95, 18.73)	<0.001
	Not available	4.11 (2.63, 6.42)	<0.001	3.08 (1.96, 4.85)	<0.001	3.97 (2.48, 6.35)	<0.001	3.05 (1.89, 4.92)	<0.001
Histology			0.002		<0.001		0.012		0.004
	Ductal carcinoma in situ	1.00 (reference)	-	1.00 (reference)	-	1.00 (reference)	-	1.00 (reference)	-
	Infiltrating ductal carcinoma	1.66 (1.12, 2.47)	0.012	2.19 (1.46, 3.28)	<0.001	1.62 (1.07, 2.44)	0.022	2.06 (1.36, 3.14)	0.001
	Lobular carcinoma	1.94 (1.11, 3.41)	0.020	2.57 (1.46, 4.54)	0.001	1.84 (1.02, 3.33)	0.044	2.43 (1.33, 4.42)	0.004
	Mucinous carcinoma	0.63 (0.24, 1.65)	0.351	1.00 (0.38, 2.61)	0.950	0.69 (0.26, 1.80)	0.442	1.03 (0.39, 2.71)	0.950
	Medullary carcinoma	0.53 (0.20, 1.38)	0.192	0.92 (0.35, 2.43)	0.871	0.57 (0.22, 1.50)	0.256	0.96 (0.36, 2.53)	0.925
	Others	1.81 (1.13, 2.89)	0.013	2.36 (1.47, 3.78)	<0.001	1.71 (1.05, 2.79)	0.033	2.16 (1.31, 3.55)	0.003
Treatment			<0.001		<0.001		<0.001		<0.001
	No treatment	1.00 (reference)	-	1.00 (reference)	-	1.00 (reference)	-	1.00 (reference)	-
	Surgery only	0.43 (0.35, 0.53)	<0.001	0.49 (0.39, 0.61)	<0.001	0.46 (0.37, 0.57)	<0.001	0.52 (0.41, 0.65)	<0.001
	Surgery with oncologic therapy	0.43 (0.34, 0.54)	<0.001	0.45 (0.35, 0.59)	<0.001	0.47 (0.36, 0.60)	<0.001	0.49 (0.37, 0.64)	<0.001
	Oncology therapy^4^	1.12 (0.89, 1.39)	0.337	0.85 (0.67, 1.07)	0.167	1.24 (0.98, 1.56)	0.077	0.92 (0.72, 1.18)	0.523

HR, hazard ratio; CI, confidence interval.

1 The forward log-rank test, backward log-rank test, and parsimonious model were applied. The proportional hazard assumption was checked and was found to have been met. Interaction between variables were checked and age was found to be a significant interaction term with ethnicity, disease stage, and histological type.

2 Simple Cox regression was applied.

3 The multiple Cox regression enter method was applied.

4 A combination of one or more therapies consisting of chemotherapy, radiotherapy, and hormonal therapy.

**Table 3.** Univariable and multivariable Cox regression analysis for prognostic factors of breast cancer related to all-cause death by age (n=2,166)

Variables		Young (<40 yr)		Middle-aged (40-59 yr)		Elderly (≥60 yr)	
		Crude HR (95% CI)^1^	Adjusted HR (95% CI)^2^	Crude HR (95% CI)^1^	Adjusted HR (95% CI)^2^	Crude HR (95% CI)^1^	Adjusted HR (95% CI)^2^
Ethnicity							
	Chinese	1.00 (reference)	1.00 (reference)	1.00 (reference)	1.00 (reference)	1.00 (reference)	1.00 (reference)
	Malay	1.72 (0.99, 3.00)^†^	1.37 (0.75, 2.48)	1.62 (1.27, 2.07)^***^	1.27 (0.98, 1.64)^†^	1.37 (1.01, 1.87)^*^	1.16 (0.85, 1.59)
	Indian	2.55 (1.10, 5.91)^*^	3.80 (1.48, 9.76)^**^	1.08 (0.70, 1.67)	0.90 (0.58, 1.41)	1.48 (1.00, 2.19)^*^	1.38 (0.93, 2.06)
	Others	0.00 (0.00, -)^4^	0.00 (0.00, -)^4^	0.65 (0.16, 2.64)	0.89 (0.22, 3.63)	0.00 (0.00, -)^4^	0.00 (0.00, -)^4^
Stage							
	0-I	1.00 (reference)	1.00 (reference)	1.00 (reference)	1.00 (reference)	1.00 (reference)	1.00 (reference)
	II	2.46 (0.29, 21.08)	1.92 (0.22,16.67)	3.08 (1.43, 6.63)^**^	3.03 (1.41, 6.53)^**^	2.04 (1.08, 3.85)^*^	1.99 (1.05, 3.75)^*^
	III	10.55 (1.36, 81.74)^*^	7.83 (1.00, 61.56)^†^	9.46 (4.50, 19.92)^***^	9.27 (4.39, 19.58)^***^	3.89 (2.03, 7.45)^***^	3.98 (2.07, 7.63)^***^
	IV	16.46 (2.09, 130.01)^**^	14.50 (1.77, 118.71)^*^	23.11 (11.08, 48.17)^***^	16.53 (7.86, 34.80)^***^	10.34 (5.54, 19.30)^***^	7.17 (3.74, 13.76)^***^
	Not available	8.30 (1.13, 60.81)^*^	6.19 (0.81, 47.06)^†^	6.14 (3.01, 12.55)^***^	4.30 (2.08, 8.87)^***^	2.35 (1.29, 4.30)^**^	1.97 (1.07, 3.65)^*^
Histology							
	Ductal carcinoma in situ	1.00 (reference)^4^	1.00 (reference)^4^	1.00 (reference)	1.00 (reference)	1.00 (reference)	1.00 (reference)
	Infiltrating ductal carcinoma	Omitted	Omitted	1.04 (0.65, 1.68)	1.33 (0.81, 2.18)	3.05 (1.50, 6.19)^**^	3.76 (1.83, 7.71)^***^
	Lobular carcinoma	Omitted	Omitted	1.13 (0.52, 2.44)	1.27 (0.58, 2.76)	3.41 (1.35, 8.65)^*^	5.10 (1.98, 13.09)^**^
	Mucinous carcinoma	Omitted	Omitted	0.16 (0.02, 1.20)^†^	0.28 (0.04, 2.09)	2.85 (0.86, 9.45)^†^	2.90 (0.86, 9.73)^†^
	Medullary carcinoma	Omitted	Omitted	0.00 (0.00, -)^4^	0.00 (0.00, -)^4^	0.00 (0.00, -)^4^	0.00 (0.00, -)^4^
	Others	Omitted	Omitted	0.84 (0.44, 1.62)	1.00 (0.52, 1.95)	4.01 (1.84, 8.75)^***^	5.44 (2.47, 11.97)^***^
Treatment							
	No treatment	1.00 (reference)	1.00 (reference)	1.00 (reference)	1.00 (reference)	1.00 (reference)	1.00 (reference)
	Surgery only	0.61 (0.31, 1.18)	0.69 (0.33, 1.41)	0.39 (0.28, 0.54)^***^	0.50 (0.36, 0.69)^***^	0.52 (0.37, 0.73)^***^	0.44 (0.31, 0.62)^***^
	Surgery with oncologic therapy	0.41 (0.18, 0.93)^*^	0.69 (0.28, 1.66)	0.40 (0.28, 0.57)^***^	0.42 (0.29, 0.60)^***^	0.63 (0.42, 0.93)^*^	0.47 (0.31, 0.70)^***^
	Oncologic therapy^3^	0.85 (0.41, 1.79)	0.87 (0.38, 2.03)	1.14 (0.82, 1.59)	0.85 (0.61, 1.19)	1.59 (1.09, 2.31)	0.87 (0.61, 1.26)

HR, hazard ratio; CI, confidence interval.

1 Simple Cox regression was applied.

2 The multiple Cox Regression enter method was applied.

3 A combination of one or more therapies consisting of chemotherapy, radiotherapy, and hormonal therapy.

4 All of the subjects were alive at the end of the study, thus the HR could not be estimated.

† p<0.1,

* p<0.05,

** p<0.01,

*** p<0.001.

**Table 4.** Univariable and multivariable Cox regression analysis for prognostic factors of breast cancer related to breast cancer-specific death by age (n=2,166)

Variables		Young (<40 yr)		Middle-aged (40-59 yr)		Elderly (≥60 yr)	
		Crude HR (95% CI)^1^	Adjusted HR (95% CI)^2^	Crude HR (95% CI)^1^	Adjusted HR (95% CI)^2^	Crude HR (95% CI)^1^	Adjusted HR (95% CI)^2^
Ethnicity							
	Chinese	1.00 (reference)	1.00 (reference)	1.00 (reference)	1.00 (reference)	1.00 (reference)	1.00 (reference)
	Malay	1.66 (0.94, 2.94)^†^	1.30 (0.70, 2.42)	1.44 (1.11, 1.87)^**^	1.12 (0.85, 1.47)	1.36 (0.98, 1.89)^†^	1.20 (0.85, 1.67)
	Indian	2.67 (1.14, 6.22)^*^	3.99 (1.53, 10.40)^**^	0.94 (0.58, 1.52)	0.79 (0.49, 1.27)	1.57 (1.05, 2.36)^*^	1.49 (0.98, 2.27)^†^
	Others	0.00 (0.00, -)^4^	0.00 (0.00, -)^4^	0.35 (0.05, 2.47)	0.47 (0.07, 3.41)	0.00 (0.00, -)^4^	0.00 (0.00, -)^4^
Stage							
	0-I	1.00 (reference)	1.00 (reference)	1.00 (reference)	1.00 (reference)	1.00 (reference)	1.00 (reference)
	II	2.47 (0.29, 21.10)	1.87 (0.22,16.30)	3.89 (1.63, 9.26)^**^	3.76 (1.58, 8.97)^**^	2.05 (1.09, 3.86)^*^	1.95 (1.03, 3.69)^*^
	III	9.64 (1.23, 75.29)^*^	6.89 (0.87, 54.71)^†^	10.29 (4.38, 24.22)^***^	10.00 (4.24, 23.60)^***^	3.72 (1.94, 7.16)^***^	3.79 (1.97, 7.30)^***^
	IV	16.60 (2.10, 131.08)^**^	15.28 (1.85, 125.98)^*^	29.60 (12.79, 68.47)^***^	20.79 (8.90, 48.57)^***^	9.13 (4.85, 17.19)^***^	6.43 (3.31, 12.51)^***^
	Not available	7.80 (1.06, 57.26)^*^	5.48 (0.72, 42.00)	7.38 (3.25, 16.78)^***^	5.28 (2.30, 12.11)^***^	1.90 (1.03, 3.50)^*^	1.63 (0.88, 3.05)
Histology							
	Ductal carcinoma in situ	1.00 (reference)^4^	1.00 (reference)^4^	1.00 (reference)	1.00 (reference)	1.00 (reference)	1.00 (reference)
	Infiltrating ductal carcinoma	Omitted	Omitted	1.05 (0.63, 1.74)	1.36 (0.80, 2.29)	2.72 (1.34, 5.54)^**^	3.01 (1.46, 6.20)^**^
	Lobular carcinoma	Omitted	Omitted	1.14 (0.50, 2.58)	1.33 (0.58, 3.02)	2.75 (1.03, 7.32)^*^	3.63 (1.34, 9.83)^*^
	Mucinous carcinoma	Omitted	Omitted	0.18, (0.02, 1.36)	0.32 (0.04, 2.41)	2.85 (0.86, 9.47)	2.62 (0.78, 8.80)
	Medullary carcinoma	Omitted	Omitted	0.00 (0.00, -)^4^	0.00 (0.00, -)^4^	0.00 (0.00, -)^4^	0.00 (0.00, -)^4^
	Others	Omitted	Omitted	0.84 (0.42, 1.69)	1.03 (0.51, 2.07)	3.49 (1.58, 7.72)^**^	4.32 (1.94, 9.65)^***^
Treatment							
	No treatment	1.00 (reference)	1.00 (reference)	1.00 (reference)	1.00 (reference)	1.00 (reference)	1.00 (reference)
	Surgery only	0.65 (0.34, 1.24)	0.63 (0.30, 1.32)	0.39 (0.29, 0.53)^***^	0.48 (0.34, 0.68)^***^	0.43 (0.31, 0.59)^***^	0.52 (0.36, 0.75)^***^
	Surgery with oncologic therapy	0.46(0.21, 1.01)^†^	0.57 (0.23, 1.42)	0.39 (0.28, 0.53)^***^	0.43 (0.29, 0.63)^***^	0.51 (0.35, 0.74)^***^	0.55 (0.36, 0.85)^**^
	Oncologic therapy^3^	0.93 (0.45, 1.92)	0.73 (0.30, 1.76)	1.03 (0.75, 1.41)	0.93 (0.65, 1.32)	1.37 (0.97, 1.94)^†^	1.00 (0.68, 1.49)

HR, hazard ratio; CI, confidence interval.

1 Simple Cox regression was applied.

2 The multiple Cox Regression enter method was applied.

3 A combination of one or more therapies consisting of chemotherapy, radiotherapy, and hormonal therapy.

4 All of the subjects were alive at the end of the study, thus the HR could not be estimated.

† p<0.1,

* p<0.05,

** p<0.01,

*** p<0.001.
